# Play attention! Therapeutic aspects to play in delirium prevention and management

**DOI:** 10.12688/wellcomeopenres.16199.1

**Published:** 2020-11-25

**Authors:** Michaela Lynn, Bethan Goulden, Meera Parmar, Paul Knopp, Michael Yeung, Ian Giles, Chloe Davies, Anadel Espinosa, Daniel Davis

**Affiliations:** 1 Department of Medicine for the Elderly, University College London Hospitals NHS Foundation Trust, London, NW1 2BU, UK; 2 Centre for Rheumatology, University College London Hospitals NHS Foundation Trust, London, NW1 2BU, UK; 3 Department of General Medicine, University College London Hospitals NHS Foundation Trust, London, NW1 2BU, UK; 4 Children and Young People’s Services, University College London Hospitals NHS Foundation Trust, London, NW1 2BU, UK; 5 MRC Unit for Lifelong Health and Ageing, Univsity College London, London, WC1E 7HB, UK

**Keywords:** Delirium, play specialists, cognitive stimulation, older adults

## Abstract

It is recognised that delirium is common among older adult inpatients and correlated with negative outcomes. The gold standard care for delirium management is achieved using multicomponent interventions. Which components work best is not yet well defined. During the COVID-19 outbreak, a paediatric ward was repurposed to treat adult patients. Paediatric nurses and play specialists remained on the ward. It was observed that the paediatric ward aesthetic and the team’s dedicated approach to cognitive stimulation and sleep promotion improved well-being among older adult patients. We propose that elements of paediatric care, primarily deployment of a play specialist, could be incorporated into a multicomponent intervention for delirium prevention and management.

## Disclaimer

The views expressed in this article are those of the author(s). Publication in Wellcome Open Research does not imply endorsement by Wellcome.

## Introduction

We are a team of multi-speciality and multi-professional staff at University College London Hospitals NHS Foundation Trust, brought together during COVID-19. Most unusually we have been treating older adult patients on a paediatric ward, staffed by paediatric nurses and a team of play specialists. While initially we believed training would mostly focus on upskilling redeployed staff, it quickly became apparent that the transfer of knowledge and skills was a two-way process and a great deal could be learned from the paediatric team, particularly in terms of delirium management as applied to adult care.

It is recognised that the gold standard care for delirium prevention and management is achieved using multicomponent interventions, including cognitive stimulation and sleep promotion. We observed that the paediatric ward aesthetic and the team’s dedicated approach to cognitive stimulation, with daily creative activities (detailed below) promoted well-being among our older adult patients. We propose that elements of their paediatric approach could be incorporated into adult delirium management, perhaps leading to a fresher, more person-centred and fun experience in hospital.

## Delirium prevention and management

Delirium is a neuropsychiatric presentation of acute illness characterised by inattention, altered arousal, cognitive impairment and psychosis. It is common, seen in 20-30% of older adult inpatients ^1^. It is associated with longer and more complicated inpatient admissions ^2^, the development and/or progression of dementia, increased institutionalisation, and mortality ^3^. Delirium also presents at younger ages, but mostly in the context of critical or post-surgical care ^4^. In children on general paediatric wards, delirium does not usually have the same implications for patient safety as it does in frail adults.

In hospital, delirium in older adults is managed by proactively screening the most vulnerable patients and by targeting risk factors that are modifiable. Many screening instruments are available and the 4AT is the one best-established within the NHS ^5, 6^.

The mainstay of delirium prevention is recognition and implementation of multicomponent, non-pharmacological interventions. These have been shown to reduce incidence of delirium by almost one third, and programmes are broadly cost-effective ^7, 8^. Nonetheless, there remains uncertainty as to which components work best ^7^. As a bundle, delirium interventions comprise a series of non-pharmacological strategies frequently carried out by nursing staff and therapists. Often, they include approaches to promote cognitive stimulation, hydration, nutrition and sleep as well as other aspects of care such as early mobilisation. Multicomponent interventions in principle are recommended by the National Institute for Health and Clinical Excellence (NICE) Delirium Clinical Guidelines (CG103), as well as the recent Scottish Intercollegiate Guidelines Network publication (SIGN 157), which state that wards should offer “a tailored multicomponent intervention package” ^9– 11^. The guidelines specifically outline a need to address cognitive impairment by introducing cognitively stimulating activities and to promote good sleep patterns and sleep hygiene. However, which strategies to adopt is not clear. This lack of evidence is acknowledged, where “the focus of future research should be...to identify the key 'active' components to improve our understanding of the determinants for successful and efficient deployment of multi‐component interventions” ^7^.

## The paediatric approach

Therapeutic play is defined as a framework of activities taking the psychosocial and cognitive development of children into account, in order to facilitate their emotional and physical well-being. Examples include games and creative activities, such as painting, sketching and storytelling. It is proven to be of high therapeutic value for ill children, contributing to both their physical and emotional well-being and to their recovery ^12^. For this reason, play specialists are an established and valued member of inpatient paediatric teams. However, they do not commonly encounter delirium and so the potential for therapeutic play in older adults has been largely unexplored.

Another common feature of paediatric wards is their aesthetic, often bright and fun. Artwork in hospitals can reduce stress for children and adults, through providing familiarity, distraction and prompts for social engagement ^13^. Conversely, it has been suggested that aesthetic deprivation, common in adult inpatient wards, might impair cognitive recovery and depress mood ^14^. Aesthetic enrichment of the clinical setting can also impact positively on staff, “promoting a clinical framework that rises above task and technique-oriented health care” ^14^.

## A unique perspective

The adult and paediatric team have been, extraordinarily, brought together by the COVID-19 outbreak. During this time, some of the normal measures to help prevent delirium, such as visits from friends and family, volunteers, musicians and therapy dogs were suspended. In addition, respiratory isolation and use of personal protective equipment by staff impaired interpersonal communication and increased sensory deprivation.

In this challenging time, we encountered numerous examples of how the paediatric team translated their skills in managing children to adults and encouraged cognitive stimulation in older adults vulnerable to delirium. In particular, staff utilised a structured approach to engage adult patients in activities including: colouring-in of images; quizzes (even via video link for those isolated in side-rooms); crosswords; sudoku; modified catch-and-throw games; themed afternoon teas, including one for ‘Victory in Europe Day’ with an appropriate era song-list; and the perennial favourite, bingo. These activities were arranged on a regular basis by a team of play specialists attached to the paediatric ward. The interventions served to reduce the isolating effects of hospitalisation during the COVID pandemic, gave patients something to look forward to each day and boosted morale. Staff also enjoyed the lively environment and enthusiastically participated in the games, quizzes and tea-parties; arguably breaking down traditional staff/patient barriers and indicating an unrecognised need for such a holistic approach to healthcare. 

Exacerbation of delirium at night is a well-known phenomenon, particularly in hospital environments and we witnessed obvious benefits from interventions common in paediatric practice being applied in this setting. For instance, successful promotion of sleep and reduced agitation occurred with the use of calming lights and soothing music in distressed, delirious patients by the paediatric nurses overnight. In addition, play specialists were available to dedicate their time to other well-being activities developed using lateral thinking, such as providing ice-lollies to help those who had stopped eating and drinking that improved oral hydration.

Patients have positively remarked on ward aesthetics; colourful murals of animals, sea and landscapes that provide distraction, decoration and a topic of conversation. It is a ward environment that feels fun, welcoming and safe as much to the children who usually occupy it as to the adults who found themselves there during a pandemic.

## Future directions

A positive output from the redeployment of adult staff and patients to a paediatric environment while caring for vulnerable older adults has been to improve patient and staff morale during the current COVID-19 pandemic. We believe that multicomponent interventions for delirium prevention in older adults may be enhanced if we can include our learning from the perspectives and experiences of health professionals not usually involved with our patient cohort. From our experiences we propose the following research agenda to investigate the effects of:

1. Incorporating a play specialist into an older adult ward for cognitive stimulation and support by quantifying changes in patient experience, patient safety, and delirium duration and severity.
2. The potential benefits of the use of calming sensory stimulation at the bedside, gentle lights and music for acutely distressed patients in order to reduce agitation, promote sleep and minimise medication use.
3. Promoting a more stimulating and interesting environment with murals and artwork throughout the ward, with an expanded role for patient input.

## Conclusions

The adage goes that "children are not just small adults". However, we suggest that acknowledging and connecting with the inner child in all of us, even as adults, has the potential to positively impact on patient outcomes. Delirium is common, and non-pharmacological approaches to prevention and treatment are heterogenous. There is currently a lack of clarity as to which strategies are most effective, though multicomponent interventions are recognised to be beneficial by various national bodies. We argue that lessons learnt from paediatric team care could be incorporated into a multicomponent intervention for delirium prevention in older adults, including the provision of a play specialist to enable a dedicated approach to engage patients in cognitively stimulating activities. There is substantial scope for this to be the focus for future research and service improvement.

## Data availability

No data are associated with this article.
