# Supplementary material for: Play attention! Therapeutic aspects to play in delirium prevention and management
Source: Wellcome Open Res. 2020 Nov 25;5:277. [Version 1] doi: 10.12688/wellcomeopenres.16199.1 (PMC7737639; doi:10.12688/wellcomeopenres.16199.1)
Supplement: Peer review history

# Peer review: Reviewer response for version 1

**DOI:** 10.21956/wellcomeopenres.17789.r41609

**Published:** 2020-12-14

**Authors:** Michael B. Fertleman

**Affiliations:** 1 Cutrale Perioperative and Ageing Group, Department of Bioengineering, Imperial College London, London, UK

This letter reports on a novel concept of older delirious patients being managed, on a presumably empty of patients, paediatric ward. Positive benefits to patients through the play therapy team were noted. In addition the bright and cheerful environment of a ward designed for children is contrasted with the classical environment of a ward for older people. 

 The authors do not mention that interactions between children and nursing home residents have been covered in the media (see https://www.theguardian.com/social-care-network/2017/sep/06/care-home-toddlers-nursery) and, through implication, perhaps the positive benefit from these older people interacting with children, is that the children were accompanied by facilitators who may have provided the stimulation to the residents.

 The team are of course to be congratulated for stepping up in the time of COVID to transfer their skills. The authors do not mention that resource allocation is different in departments of geriatric medicine. In most wards budgets are so tight that such therapy is not available, although many geriatricians would anecdotally agree that when any rare distraction arrives (carol singers, musicians, hair dressers, pets, the CEO) the atmosphere of the ward changes.

Does the article adequately reference differing views and opinions?

Yes

Are all factual statements correct, and are statements and arguments made adequately supported by citations?

Yes

Is the Open Letter written in accessible language?

Yes

Where applicable, are recommendations and next steps explained clearly for others to follow?

Yes

Is the rationale for the Open Letter provided in sufficient detail?

Yes

Reviewer Expertise:

Perioperative care

I confirm that I have read this submission and believe that I have an appropriate level of expertise to confirm that it is of an acceptable scientific standard.

# Peer review: Reviewer response for version 1

**DOI:** 10.21956/wellcomeopenres.17789.r41606

**Published:** 2020-11-26

**Authors:** Roman Romero-Ortuno

**Affiliations:** 1 Discipline of Medical Gerontology, Mercer's Institute for Successful Ageing, St James's Hospital, Dublin, Ireland; 2 Global Brain Health Institute, Dublin, Ireland

Thank you for asking me to review this very interesting article. Among gerontological clinical practitioners, it is well known that the characteristics and quality of the healthcare environment can be of extreme relevance for the management of delirium, which is a common occurrence in hospitalised older adults and has very negative, often underappreciated, consequences for patients, their loved ones, and staff.

 It is often said that truly inclusive environments need to be able to cater for the most vulnerable and/or special, and when they do everyone else's needs become then 'automatically' covered. Therefore, such approaches have the potential to introduce large-scale quality improvement in organisations.

 There are two other very important themes in this letter; a) the intergenerational approach and b) the interdisciplinary approach to care. Both are of increasing relevance and require evidence generation as per authors' future directions.

Does the article adequately reference differing views and opinions?

Yes

Are all factual statements correct, and are statements and arguments made adequately supported by citations?

Yes

Is the Open Letter written in accessible language?

Yes

Where applicable, are recommendations and next steps explained clearly for others to follow?

Yes

Is the rationale for the Open Letter provided in sufficient detail?

Yes

Reviewer Expertise:

Ageing, Brain Health

I confirm that I have read this submission and believe that I have an appropriate level of expertise to confirm that it is of an acceptable scientific standard.
